# Editorial: Methods, applications, and protocols in plant science: Network modeling-guided understanding of gene regulation in plants

**DOI:** 10.3389/fpls.2023.1171846

**Published:** 2023-03-07

**Authors:** Dae Kwan Ko, M. Teresa Sanchez-Ballesta

**Affiliations:** ^1^ MSU-DOE Plant Research Lab, Michigan State University, East Lansing, MI, United States; ^2^ Department of Plant Biology, Michigan State University, East Lansing, MI, United States; ^3^ Great Lakes Bioenergy Research Center, Michigan State University, East Lansing, MI, United States; ^4^ Department of Characterization, Quality, and Safety, Institute of Food Science, Technology and Nutrition (ICTAN-CSIC), Ciudad Universitaria, Madrid, Spain

**Keywords:** gene regulation, multi-omics, gene networks, plant growth, plant development, abiotic stress

Like our daily lives with social network connections in various forms, molecular components (e.g., DNA, RNA and proteins) interact with each other to create biological networks, which allow complex organisms to timely coordinate appropriate biological responses. Surowiecki mentioned “the wisdom of crowds”, which refers to the phenomenon in which the collective knowledge of a community is greater than the knowledge of any individual. Similarly, if gene responses to internal or external stimuli could be investigated at “a network level” in plants, their function could be better understood and enabling for crop improvement. Network-enabled understanding of biological questions regarding a broad array of Research Topics including plant development, growth, and responses to environmental stress requires the ability to obtain genome-wide molecular measurements, which has been possible by the ever-increasing access to and decreasing costs of next-generation sequencing technology over the past decade. Thus, in the post-genomic era, gene network and omics approaches have been more powerful in the field of plant biology than ever before. In this Research Topic *Methods*, *Applications, and Protocols in Plant Science: Network Modeling-guided Understanding of Gene Regulation in Plants*, the collection of research articles and reviews highlights recent progress in applying gene network or omics approaches to understanding gene expression changes necessary for significant agronomic traits. We believe that this broad snapshot would advance this inspiring field to the next level and provide opportunities for readers to spark new exciting ideas in their research.

Cotton is not only the most significant source of natural fiber worldwide but also a powerful model for understanding plant architecture. Two cultivated worldwide allotetraploid species, *Gossypium hirsutum* (Upland cotton) and *G. barbadense* (Sea-island cotton), have complex branching patterns mediated by complex gene regulatory networks. The review presented by Huang et al. takes a deep dive into the genetic basis underlying cotton plant architecture QTLs and the regulatory signaling pathways, the underlying molecular factors, and the evolutionary and bioengineering perspectives.

Gene homologs descend from a common evolutionary origin. Intriguingly, some genes have no sequence similarity with genes in other species. Those genes are referred to as orphan genes (OGs) and are ubiquitous in the genomes of sequenced organisms. In plants, OGs are associated with lineage-specific evolution, responses to environmental stress and metabolisms. Jiang et al. present current advances in understanding the functional roles of OGs, focusing on the identification of OGs, gene expression modes and the association with significant biological traits. Possible future direction for functional studies of OGs is discussed.

Climate change, whose frequency and severity are projected to increase, challenges plant growth, development, and crop productivity worldwide. To withstand these stressors, plants have evolved highly interconnected regulatory networks that coordinate proper cellular and metabolic responses. Epigenetic control is one of the elaborate mechanisms in these regulatory efforts. Lancikova et al. report the identification of cytosine-5 DNA methyltransferase and demethylase genes in Amaranth (*Amaranthus cruentus* L.), and further, delineate their functional roles in response to heavy metal stress by exploring the gene expression profiles in different stress conditions and tissues. This finding may help us to characterize the molecular mechanism by which these epigenetic components regulate the dynamic gene expression changes under heavy metal stress in Amaranth and related crops.

Brhane et al. characterize transcriptome profiles in response to aluminum toxicity in finger millet, which is a widely grown cereal in arid and semi-arid areas in Africa and Asia. Through a large-scale transcriptome assembly, this study presents high-quality genomics resources covering 198,546 unique genes, 3,553 single-copy SSR markers, and 119,073 SNP markers. The transcriptome profiling reveals hundreds of differentially expressed genes in genotypes tolerant to aluminum toxicity along with their annotated function. The genomic resources obtained in this study will serve as a fundamental resource for the research communities.

Taken together, the Research Topic illustrates the potential derived from gene network and omics approaches in the field of plant biology. With these research articles and reviews, this Research Topic would provide new genomics resources and deep insights into the application of these approaches for crop improvement.

## Author contributions

DKK wrote the draft. MTS-B edited the text. All authors approve the submission.

